# *In Silico* Mining of the Streptome Database for Hunting Putative Candidates to Allosterically Inhibit the Dengue Virus (Serotype 2) RdRp

**DOI:** 10.3390/ph18081135

**Published:** 2025-07-30

**Authors:** Alaa H. M. Abdelrahman, Gamal A. H. Mekhemer, Peter A. Sidhom, Tarad Abalkhail, Shahzeb Khan, Mahmoud A. A. Ibrahim

**Affiliations:** 1 Computational Chemistry Laboratory, Chemistry Department, Faculty of Science, Minia University, Minia 61519, Egypt; 2 Department of Pharmaceutical Chemistry, Faculty of Pharmacy, Tanta University, Tanta 31527, Egypt; 3 Department of Botany and Microbiology, College of Science, King Saud University, P.O. Box 2455, Riyadh 11451, Saudi Arabia; 4 Centre for Pharmaceutical Engineering Science, Faculty of Life Science, School of Pharmacy and Medical Sciences, University of Bradford, Bradford BD7 1DP, UK; 5 Department of Engineering, College of Engineering and Technology, University of Technology and Applied Sciences, Nizwa 611, Oman; 6 School of Health Sciences, University of KwaZulu-Natal, Westville Campus, Durban 4000, South Africa

**Keywords:** Dengue virus, RdRp, Streptome database, virtual screening, molecular dynamics simulations, quantum mechanical computations

## Abstract

**Background/Objectives:** In the last few decades, the dengue virus, a prevalent flavivirus, has demonstrated various epidemiological, economic, and health impacts around the world. Dengue virus serotype 2 (DENV2) plays a vital role in dengue-associated mortality. The RNA-dependent RNA polymerase (RdRp) of DENV2 is a charming druggable target owing to its crucial function in viral reproduction. In recent years, streptomycetes natural products (NPs) have attracted considerable attention as a potential source of antiviral drugs. **Methods:** Seeking prospective inhibitors that inhibit the DENV2 RdRp allosteric site, *in silico* mining of the Streptome database was executed. AutoDock4.2.6 software performance in predicting docking poses of the inspected inhibitors was initially conducted according to existing experimental data. Upon the assessed docking parameters, the Streptome database was virtually screened against DENV2 RdRp allosteric site. The streptomycetes NPs with docking scores less than the positive control (**68T**; calc. −35.6 kJ.mol^−1^) were advanced for molecular dynamics simulations (MDS), and their binding affinities were computed by employing the MM/GBSA approach. **Results:** SDB9818 and SDB4806 unveiled superior inhibitor activities against DENV2 RdRp upon MM/GBSA//300 ns MDS than **68T** with Δ*G*_binding_ values of −246.4, −242.3, and −150.6 kJ.mol^−1^, respectively. A great consistency was found in both the energetic and structural analyses of the identified inhibitors within the DENV2 RdRp allosteric site. Furthermore, the physicochemical characteristics of the identified inhibitors demonstrated good oral bioavailability. Eventually, quantum mechanical computations were carried out to evaluate the chemical reactivity of the identified inhibitors. **Conclusions:** As determined by *in silico* computations, the identified streptomycetes NPs may act as DENV2 RdRp allosteric inhibitors and mandate further experimental assays.

## 1. Introduction

The dengue virus (DENV) is categorized within the *Flaviviridae* family and is part of the Orthoflavivirus genus [1]. The Orthoflavivirus genus also involves other viruses, like Zika and West Nile, that transmit human diseases through ticks and mosquitoes [2,3]. Typically, DENV is transferred to individuals by bites from female mosquitoes, especially A. aegypti [4]. It has been recently reported that DENV is responsible for more than 25,000 fatalities and 100 million sicknesses every year throughout the world, indicating that the DENV catastrophe is having a significant impact on global healthcare [5,6,7]. The earliest known serotype of DENV was DENV1, which was isolated for the first time in 1943 [8]. Dengue contagion is induced by four types of viruses (DENV1–4) that participate in approximately 65% of their genome [8]. In spite of this genetic resemblance, each serotype still exhibits significant genetic discrepancy [9]. Among the serotypes, DENV2 plays a key function in dengue-associated mortality, as it is not only described by its specific antigenic properties but also shares similarities with other types [10]. The genetic makeup of DENV2 consists of an 11-kilobase RNA molecule [11]. DENV2 RNA is surrounded by open reading frames (ORFs) as well as 5′ and 3′ untranslated regions (UTRs) that are important for translation, replication, and packaging [12]. In spite of the serious repercussions and high mortality rates of the DENV2 contagion, particular therapeutic choices other than symptomatic relief remain scarce. This challenge is caused by a variety of factors, including DENV2’s rapid replication, repeated alterations, and the utilization of multiple strategies to pervert the host immune system [13,14,15]. Considering these challenges, continuous research on curative techniques for DENV2 contagion is specifically complicated.

Furthermore, about three structural proteins, including envelope, membrane, and capsid protein, as well as seven nonstructural proteins (NSP1–5), are encoded by the DENV2 genomic constituent [16]. These components make up the external sheath of DENV2, which is essential for interaction with the host cell and the escape of immune ripostes [17,18]. Of note, each of the seven NSPs plays a distinct role. In addition to viral repetition, NSP1 also contributes to immune evasion, while NSP2A participates in viral congregation and RNA replication [19]. The NSP2B works as a cofactor and is necessary for the protease activity and viral replication of NSP3 [19]. NSP4A expedites host cell membrane realignment and viral reproduction, whereas NSP4B pitches in forming viral repetition complexes and regulates the host immune retorts [20]. NSP5 acts as an RNA-dependent RNA polymerase (RdRp) and methyltransferase and is crucial for viral RNA synthesis and capping [21]. RdRp is in charge of both positive- and negative-strand RNA synthesis through the replication and transcription of its genome [22,23,24]. There is 65–70% sequence homology among the four DENV serotypes [25]. Due to the absence of a mammalian equivalent for DENV RdRp, this offers an appealing chance for discovering effective and novel antiviral medicines [26,27]. Recently, DENV2 RdRp has attracted increasing attention, and many RdRp inhibitors have been proposed and investigated [28,29,30,31,32]. RdRp inhibitors typically target two fundamental kinds of binding sites, namely, the allosteric and the catalytic binding sites [33]. Nucleoside inhibitors (NIs), including remdesivir, favipiravir, BCX4430, and ribavirin, demonstrate wide-spectrum antiviral activity via inhibiting the RdRp binding site [34,35,36,37]. NIs are phosphorylated into triphosphate analogs, which causes them to integrate into the growing viral RNA strand, which, in turn, terminates the RNA replication process. Nevertheless, the inhibition mechanism employed by NIs frequently results in off-target side effects [38]. Conversely, non-nucleoside inhibitors (NNIs) bind to the RdRp allosteric site and demonstrate antiviral activity by inhibiting the configurational changes necessary for viral RNA transcription [39]. NNIs have attracted significant interest in antiviral medication evolution because of their low toxicity and low side effects [40]. A promising allosteric inhibitor of DENV2 RdRp has been identified by the Novartis Institute for Tropical Diseases, namely an 8-quinolyl sulfonamide (**27/68T**), demonstrating significant activity against all DENV serotypes with an average IC_50_ value ranging from 0.013 to 0.074 μM [41]. However, this compound did not succeed in safety or effectiveness evaluations in clinical trials [42]. Consequently, the discovery of effective drug candidates that inhibit the DENV2 RdRp remains a formidable challenge.

**Figure 1:**
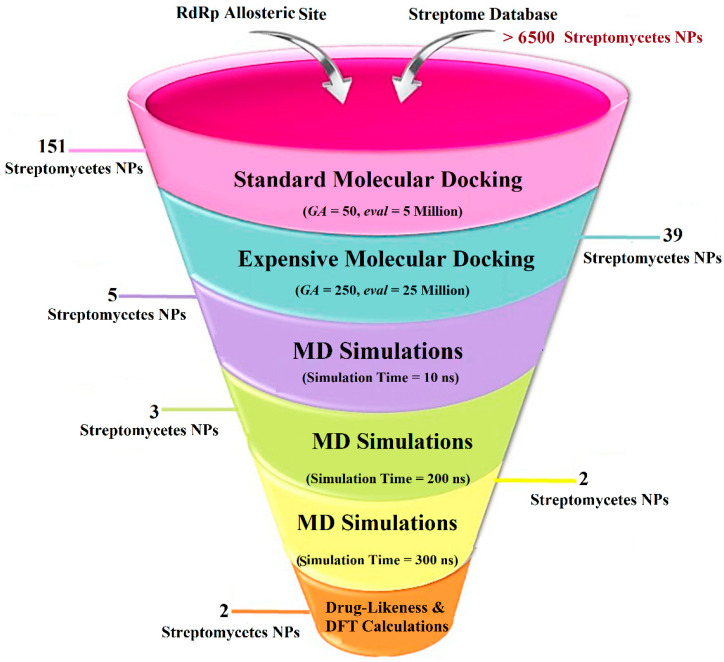
Virtual screening workflow for identifying allosteric DENV2 RdRp inhibitors from the Streptome database using various *in silico* approaches.

Streptomyces have been recognized as an important source of medicinal drugs [43]. Precisely, the *Streptomyces* species is known to produce over two-thirds of clinically used antibiotics and other pharmacologically important compounds [44]. Streptomycetes have demonstrated potential antiviral activity by targeting various viral enzymes and proteins, including virantmycin and alanosine [45]. Narasin, an ionophore isolated from *Streptomyces aureofaciens*, has been found to inhibit the replication of DENV [46]. It has been documented that the use of narasin as a treatment for DENV2-infected cells from 12 to 48 h after infection did not decrease the levels of both positive-strand and negative-strand DENV2 RNA, suggesting that narasin does not impede DENV2 RNA replication [46,47]. Unlike broader natural product libraries (e.g., ZINC Natural Products, NPASS, or SuperNatural II) that include a wide range of compounds from diverse sources, the Streptome database uniquely provides the most comprehensive collection of streptomyces-derived natural products [48]. This enhances the likelihood of identifying biologically active scaffolds with established antimicrobial or therapeutic potential. Seeking effective anti-DENV2 drug candidates, the Streptome database, containing > 6500 streptomycetes natural products (NPs), was mined to hunt novel allosteric inhibitors toward DENV2 RdRp with a higher potency than **68T**. Upon the docking computations, the most potent streptomycetes NPs were picked up and advanced for molecular dynamics simulations (MDS) over 300 ns, accompanied by binding energies utilizing the MM/GBSA approach. Post-MD analyses were executed on the most promising streptomycetes NPs bound to the DENV2 RdRp. Additionally, the drug-like features of the identified streptomycetes NPs were predicted. Furthermore, quantum mechanical computations were employed to obtain a deeper understanding of the geometrical and electronic properties of the identified streptomycetes NPs. A schematic diagram of the applied computational approaches to filter the Streptome database is portrayed in Figure 1. The obtained results highlight the potential of the identified streptomycetes NPs as allosteric DENV2 RdRp inhibitors and offer viable curative candidates for future experimental assays.

## 2. Results and Discussion

### 2.1. Docking Protocol Assessment

**Figure 2:**
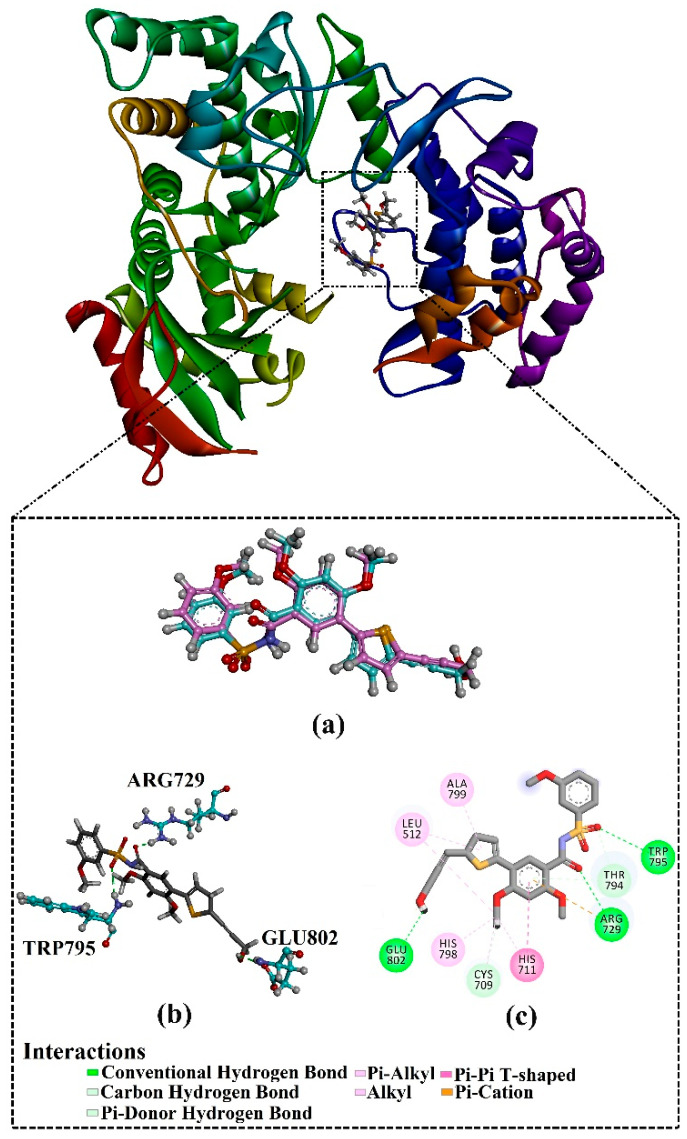
(**a**) Overlapping between the original binding pose (pink) and the predicted docking pose (cyan) and (**b**) 3D and (**c**) 2D illustrations of the predicted binding pose of **68T** against DENV2 RdRp allosteric site.

Prior to data generation, the AutoDock4.2.6 software’s performance in predicting the binding pose of the co-crystallized compound **27/68T** inside the DENV2 RdRp allosteric site was validated based on the available experimental data. The anticipated binding pose of compound **27/68T** was compared to its original binding pose (PDB entry: 5K5M [41]) (Figure 2). According to the literature, the RMSD values between the anticipated binding pose and the resolved original binding pose should be <2.0 Å [49,50,51]. From Figure 2, the predicted pose closely matched the experimental structure with an RMSD value of 0.79 Å. Examining the binding pose of **68T** demonstrated three fundamental H-bonds with ARG729 (3.11 Å), TRP795 (3.40 Å), and GLU802 (2.67 Å). As well, the **68T** exhibited a pi-pi T-shaped interaction with HIS711 and a pi-cation interaction with ARG729. Re-docking results showed that the AutoDock2.4.6 software correctly predicted the binding pose of ligand-RdRp complexes. As a result, the AutoDock4.2.6 software was used to mine the Streptome database for hunting putative DENV2 RdRp ligands.

### 2.2. Virtual Screening of the Streptome Database

**Figure 3:**
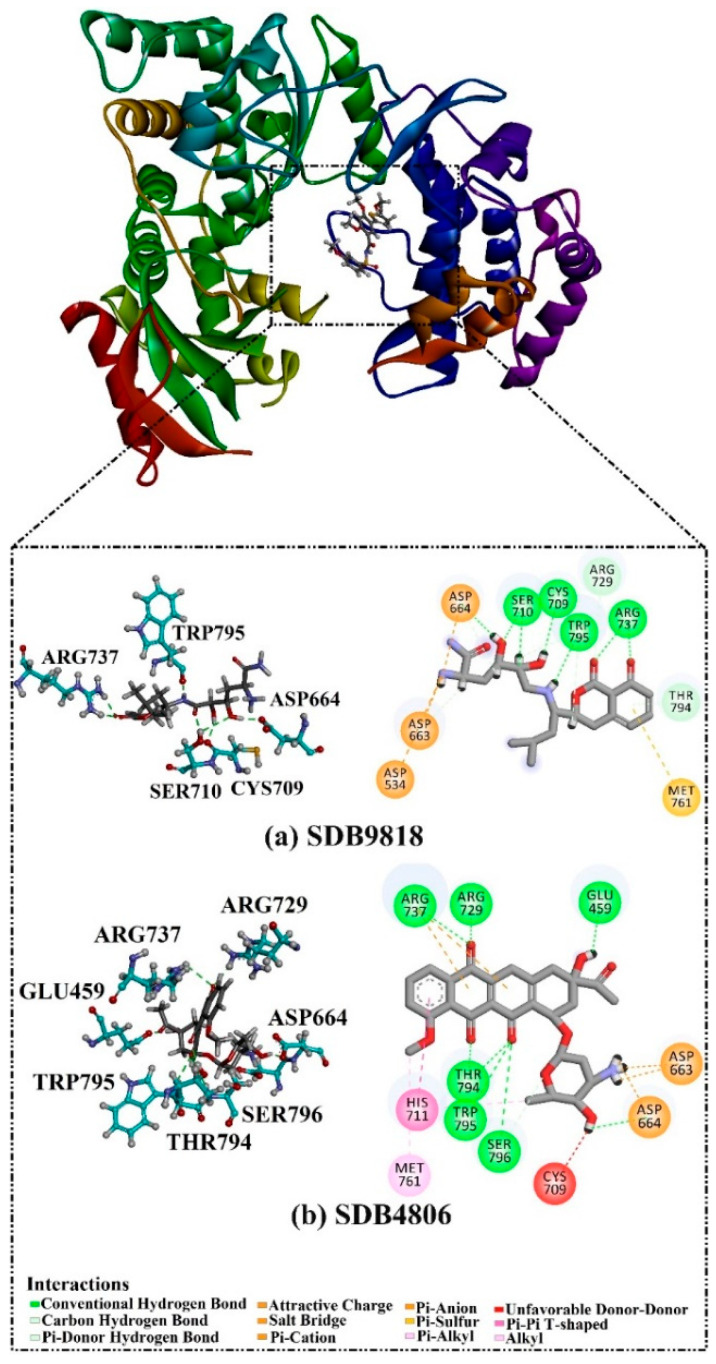
3D and 2D Illustrations of the predicted docking poses of (**a**) SDB9818 and (**b**) SDB4806 inside the DENV2 RdRp allosteric site.

A virtual screening technique can be used to hunt prospective bioactive inhibitors at the early stages of the drug design process [52]. Herein, the Streptome database encompassing > 6500 NPs was mined using standard docking parameters of *eval* = 5 million and *GA* = 50. On the basis of standard docking computations, only 151 streptomycetes NPs unveiled docking scores equal to or lower than that of **68T** (calc. −35.6 kJ.mol^−1^). As a result, these 151 streptomycetes NPs were re-docked toward the DENV2 RdRp utilizing expensive parameters (i.e., *eval* = 25 million and *GA* = 250) (Table S1). As reported in Table S1, only 39 streptomycetes NPs revealed lower docking scores in comparison with **68T** (calc. −35.6 kJ.mol^−1^). Figure S1 depicts the 2D illustrations for the anticipated binding poses of these 39 streptomycetes NPs within the DENV2 RdRp allosteric site. As shown in Figure S1, all investigated streptomycetes NPs within the DENV2 RdRp allosteric site displayed a fundamental H-bond with ARG729 and TRP795. Pi-Sigma, pi-pi T-shaped, amide-pi stacked, carbon H-bond, and pi-cation interactions were also monitored between the inspected streptomycetes NPs and the main residues in the DENV2 RdRp allosteric site. 2D Chemical structures, computed standard and expensive docking scores, and the intramolecular H-bond of the top 10 scoring streptomycetes NPs toward DENV2 RdRp are registered in Table 1. As well, the 3D and 2D depictions for the predicted binding poses of two outstanding streptomycetes NPs within the DENV2 RdRp allosteric site are illustrated in Figure 3. Furthermore, the most potent two streptomycetes NPs were re-docked using *GA* = 1000 against the DENV2 RdRp allosteric site. Comparing results obtained with a *GA* value of 250 to those using a *GA* value of 1000, no notable differences were observed in the docking poses and scores of these two streptomycetes NPs within the DENV2 RdRp allosteric site (Figure S2). Of note, these two promising streptomycetes NPs were selected according to the evaluated binding affinity throughout 300 ns MDS, as evidenced in the MDS section.

According to data listed in Table 1, SDB9818 manifested a significantly favorable docking score of −46.9 kJ.mol^−1^ against the DENV2 RdRp allosteric site. SDB9818 is a natural product isolated from *Streptomyces* sp. VITBRK2, which shows a high activity against MRSA [53]. Structural insights into the docking pose of SDB9818 against the DENV2 RdRp allosteric site showed that the CO of SDB9818 formed three H-bonds with the NH_2_ of ARG737 (2.04, 2.62, and 1.83 Å). Moreover, the two OH groups of (2*R*,3*S*)-butane-2,3-diol exhibited three H-bonds with the OH of SER796 (3.01 Å), CO of CYS709 (1.83 Å), and carboxylate of ASP664 (1.74 Å). Ultimately, the NH and CO groups of formamide showed two H-bonds with the CO of TRP795 (1.80 Å) and the OH of SER710 (1.70 Å) (Figure 3).

SDB4806 is an anthracycline antibiotic extracted from *Streptomyces peucetius* var. *aureus*, which demonstrates anticarcinoma activity by impeding RNA and DNA synthesis [54]. SDB4806 revealed the second-lowest docking score of −45.6 kJ.mol^−1^ against the DENV2 RdRp allosteric site. According to Figure 3, SDB4806 displayed eight H-bonds with the essential residues of the DENV2 RdRp allosteric site. More precisely, the OH groups of SDB4806 established two H-bonds with the carboxylate of GLU459 (1.63 Å) and ASP664 (1.84 Å). Moreover, the CO groups of SDB4806 exhibited six H-bonds with the NH_2_ of ARG729 (2.16 Å), NH_2_ of ARG737 (2.84 Å), OH of THR794 (1.97 and 2.44 Å), NH of TRP795 (2.54 Å), and NH of SER796 (2.83 Å).

According to sequence and structure comparisons, the DENV2 RdRp allosteric site is not homologous to any known domains in human DNA/RNA polymerases or bacterial polymerases, reducing off-target interactions [55]. Moreover, SDB9818 and SDB4806 often do not exhibit significant activity against eukaryotic polymerases unless they are specifically selected or designed to have this effect.

### 2.3. Molecular Dynamics Simulations (MDS)

**Figure 4:**
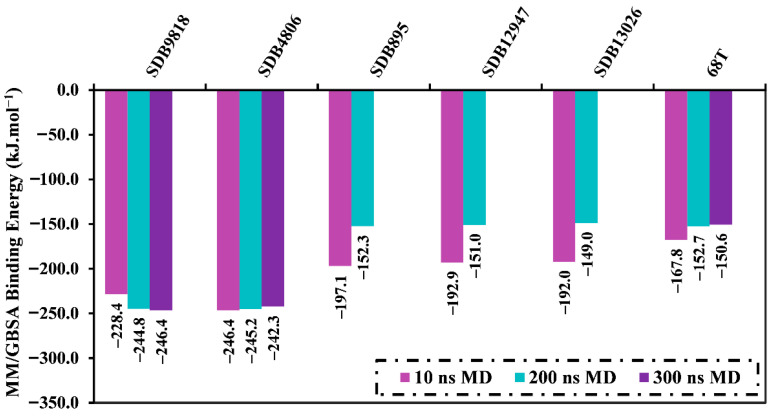
Estimated binding energies for SDB9818, SDB4806, SDB895, SDB12947, SDB13026, and **68T** bound to the DENV2 RdRp allosteric site over 10, 200, and 300 ns MDS.

To examine receptor–ligand stability and conformational variations, MDS has been widely used to analyze the dynamic properties of the inhibitor complexed with receptors. The most promising 39 streptomycetes NPs with docking scores less than **68T** (calc. −35.6 kJ.mol^−1^) in the complex with the DENV2 RdRp allosteric site were advanced for MDS over 10 ns. Table S2 lists the corresponding MM/GBSA binding energies over the 10 ns MDS. As found in Table S2, only five streptomycetes NPs unveiled higher binding affinities than **68T** (Δ*G*_binding_ = −167.8 kJ.mol^−1^) toward the DENV2 RdRp allosteric site. In order to achieve more reliable outcomes, these five streptomycetes NPs in complex with the DENV2 RdRp allosteric site were introduced to longer MDS throughout 200 ns, accompanied by MM/GBSA binding energy estimations (Figure 4). As depicted in Figure 4, two streptomycetes NPs, namely SDB9818 and SDB4806, demonstrated lower binding energies compared to **68T** (Δ*G*_binding_ = −152.7 kJ.mol^−1^). Moreover, a 300 ns MDS was executed for these two streptomycetes NPs bound to the DENV2 RdRp allosteric site, followed by binding affinity computations (Figure 4). From Figure 4, it is obvious that no significant variations were observed in the evaluated binding energies over 200 and 300 ns MDS for the identified two streptomycetes NPs complexed with the DENV2 RdRp allosteric site. In comparison with **68T** (Δ*G*_binding_ = −150.6 kJ.mol^−1^), SDB9818 and SDB4806 unveiled superior binding affinities toward the DENV2 RdRp allosteric site throughout 300 ns MDS with Δ*G*_binding_ values of −246.4 and −242.3 kJ.mol^−1^, respectively. Moreover, the standard deviation and standard error of the mean for the binding energy (Δ*G*_binding_) of the two identified streptomycetes NPs and **68T** complexed with the DENV2 allosteric site over 300 ns MDS were computed (Table S3). As registered in Table S3, the low standard deviation and standard error of mean values indicated that the binding energy remained relatively stable throughout the simulation time. These outcomes illuminated the efficiency of the identified streptomycetes NPs as promising anti-DENV2 drug candidates.

The main limitation of the current research is the lack of MDS runs replication for the identified streptomycetes NPs as promising anti-DENV2 drug candidates. As a consequence, the future study will consider multiple independent MDS with a variety of initial velocities in order to verify the consistency of the findings.

**Figure 5:**
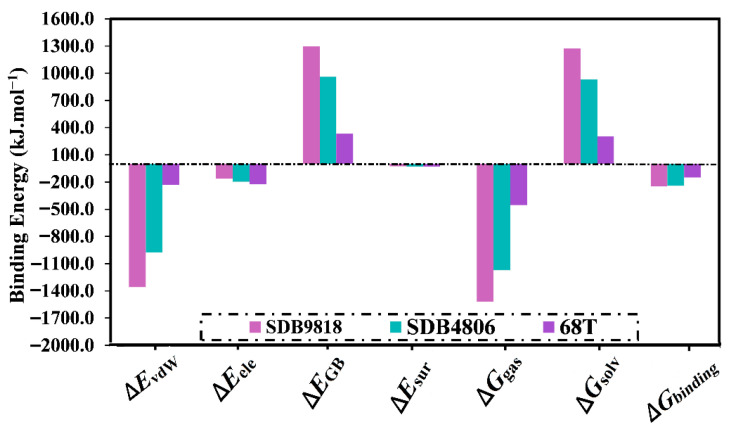
The estimated separate items of the binding energies for SDB4806, SDB9818, and **68T** bound to the DENV2 RdRp allosteric site over 300 ns MDS.

The decomposition of the computed binding energies into separate components was then executed and is depicted in Figure 5. As depicted in Figure 5, the *E*_vdW_ was the predominant participation in the Δ*G*_binding_ of SDB9818, SDB4806, and **68T** complexed with the DENV2 RdRp allosteric site, with mean values of −1358.5, −977.4, and −228.9 kJ.mol^−1^, respectively. Moreover, the *E*_ele_ demonstrated a favorable contribution to Δ*G*_binding_ with the mean values of −160.7, −196.2, and −224.3 kJ.mol^−1^ for SDB4806, SDB9818, and **68T** complexed with the DENV2 RdRp allosteric site, respectively.

**Figure 6:**
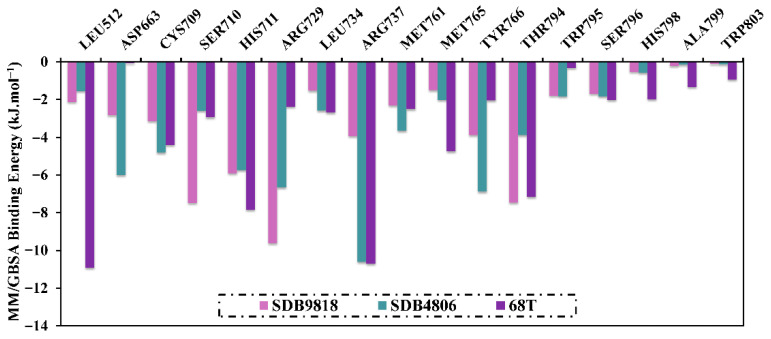
Per-residue energy decomposition analysis of SDB9818-, SDB4806-, and **68T**-RdRp complexes throughout 300 ns MDS.

To obtain more insights into the fundamental amino acids included in the inhibition of the DENV2 RdRp allosteric site, the per-residue energy decomposition was performed and plotted in Figure 6. It is worth noting that only residues with Δ*G*_binding_ < −2.1 kJ.mol^−1^ were taken into account. From the delineated per-residue energy decomposition in Figure 6, ARG729, THR794, TRP795, SER796, and ARG737 disclosed promising participations in the binding of SDB9818, SDB4806, SDB895, and **68T** with the allosteric site of the DENV2 RdRp. The significant participation of ARG729 in the allosteric site of DENV RdRp evinced −9.6, −6.7, −7.1, and −2.5 kJ.mol^−1^ for SDB9818-, SDB4806-, and **68T**-RdRp complexes, respectively.

### 2.4. Post-MD Analyses

In order to further investigate the steadiness and behavior of SDB9818-, SDB4806-, and **68T**-RdRp, post-MD analyses were accomplished over the course of 300 ns MDS. Six properties were evaluated, including root-mean-square fluctuation and deviation (RMSF and RMSD), radius of gyration (Rg), binding energy per trajectory, H-bond analysis, and solvent-accessible surface area (SASA).

#### 2.4.1. Binding Energy per Trajectory

**Figure 7:**
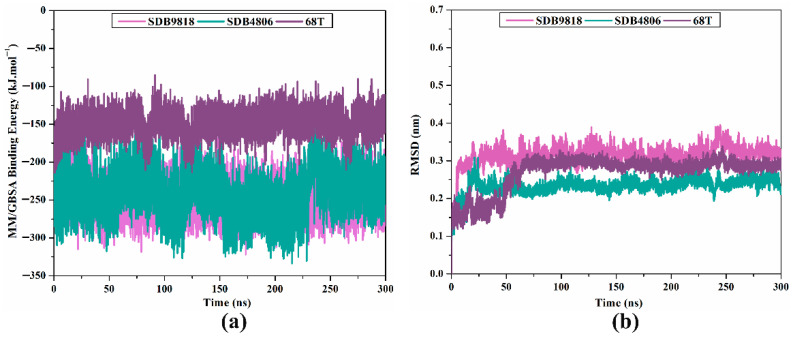
(**a**) Binding energy per trajectory and (**b**) RMSD of SDB9818 (pink), SDB4806 (cyan), and **68T** (dark mauve) bound to the DENV2 RdRp allosteric site throughout the 300 ns MDS.

The energetic persistence of SDB9818, SDB4806, and **68T** bound to the DENV2 RdRp allosteric site was evaluated by measuring the binding energy versus time correlation (Figure 7a). As illustrated in Figure 7a, the comprehensive constancy for SDB9818, SDB4806, and **68T** bound to the DENV2 RdRp allosteric site was observed with average Δ*G*_binding_ values of −246.4, −242.3, and −150.6 kJ.mol^−1^, respectively. The most striking outcome to emerge from this graph is that all inspected complexes conserved steadiness throughout 300 ns MDS.

#### 2.4.2. RMSD Analysis

In order to supply valuable insights into ligand–receptor structural steadiness, RMSD for the backbone atoms relative to the initial position was measured over 300 ns MDS (Figure 7b). Based on Figure 7b, the mean RMSD values were 0.32, 0.23, and 0.27 nm for SDB9818-, SDB4806-, and **68T**-RdRp complexes, respectively. These results demonstrated that these inspected streptomycetes NPs were bound tightly and were overall stable within the RdRp allosteric site. Furthermore, multiple snapshots taken at consistent intervals throughout the MDS were extracted to further assess the stability of the identified streptomycetes NPs within the DENV2 RdRp allosteric site. The 3D binding patterns of the identified streptomycetes NPs inside the allosteric site of DENV2 RdRp are depicted in Figure S3. As illustrated in Figure S3, the identified streptomycetes NPs sustained stable interactions at the allosteric site of streptomycetes NPs, resulting in the formation of a stable RdRp-inhibitor complex.

#### 2.4.3. Rg Analysis

**Figure 8:**
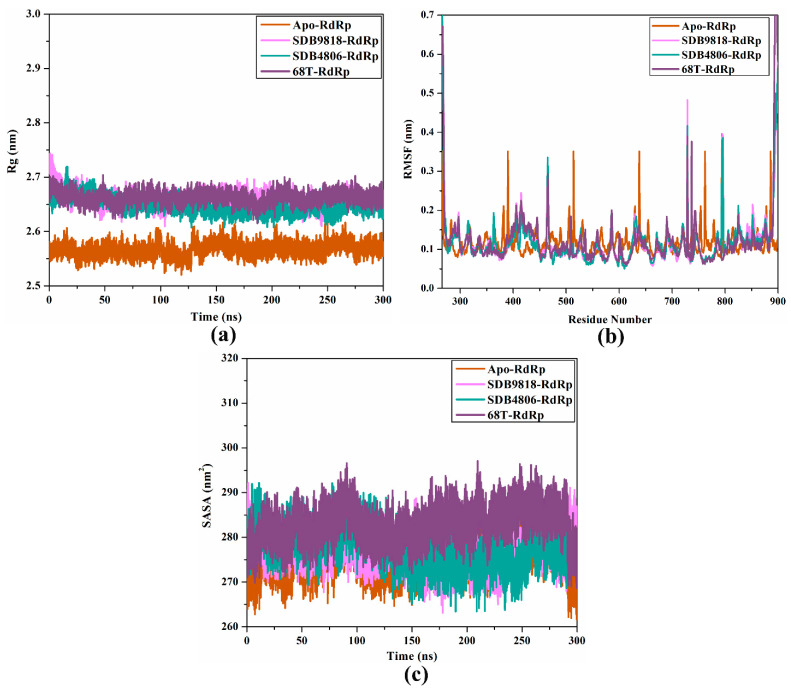
(**a**) Rg, (**b**) RMSF for the backbone atoms, and (**c**) SASA analyses for apo-RdRp (orange), SDB9818-RdRp (pink), SDB4806-RdRp (cyan), and **68T**-RdRp (dark mauve) over 300 ns MDS.

The Rg is one of the parameters utilized to gauge the equilibrium configurations of the whole system, and this assists in determining receptor compactness during MDS. Protein compactness is dependent on their residue sequence constitution, and it changes during receptor–ligand interactions [56]. As a result, the Rg for SDB4806, SDB9818, and **68T** complexed with the DENV2 RdRp allosteric site was estimated throughout 300 ns MDS in order to investigate how residue flexibility affects enzyme compactness (Figure 8a). The measured average Rg values were 2.57, 2.66, 2.65, and 2.66 nm for apo-, SDB4806-, SDB9818-, and **68T**-RdRp, respectively. These results implied that the RdRp structure was well-compacted and more steady after the complexation with SDB4806 and SDB9818.

#### 2.4.4. RMSF Analysis

RMSF is a parameter utilized for determining the elasticity of every residue of the target during the MDS. RMSF explains variations in the structural configurations of the target from the starting position until the termination of the simulations according to the residue fluctuations [57]. Thus, the RMSF was evaluated over 300 ns MDS to grasp how structural deviations affect target elasticity (Figure 8b). It is known that greater RMSF demonstrates higher elasticity of the receptor during simulations. As illustrated in Figure 8b, it was noticed that the **68T**-RdRp had a greater appearance of oscillating residues compared to the SDB4806 and SDB9818 complexed with the DENV2 RdRp allosteric site. The average RMSF values were 0.117, 0.118, 0.124, and 0.122 nm for apo-, SDB9818-, SDB4806-, and **68T**-RdRp, respectively. Based on RMSF results, most of the residues of the DENV2 RdRp allosteric site exhibited higher stability after complexation with SDB4806, SDB9818, and **68T**.

#### 2.4.5. SASA Analysis

Water plays a substantial role in the determination of the stability, structure, role, and dynamics of targets. SASA is an indicator that is utilized for measuring the attainability of protein amino acids to encompass water molecules, and any alterations in the attainability could impact protein functions, dynamics, and structure [58]. Herein, SASA was employed to gauge the binding impact of the investigated inhibitor on the configurational behavior of the DENV2 RdRp allosteric site. Figure 8c illustrates the SASA of the DENV2 RdRp allosteric site upon binding with SDB4806, SDB9818, and **68T** throughout 300 ns MDS. As can be seen from the SASA plots, SDB4806, SDB9818, and **68T** demonstrated similar frame patterns after binding to the RdRp allosteric site. Nevertheless, the SASA of RdRp increased upon binding with the **68T** (Figure 8c). In contrast, as SDB4806 and SDB9818 were bound to the RdRp allosteric site, the SASA value slightly decreased. The average SASA values were 275.9, 277.0, 277.8, and 282.9 nm^2^ for apo-, SDB4806-, SDB9818-, and **68T**-RdRp, respectively. SASA outcomes indicated that the streptomycetes NPs and **68T** had little impact on the solvent accessibility of the DENV2 RdRp enzyme.

#### 2.4.6. H-Bond Analysis

**Figure 9:**
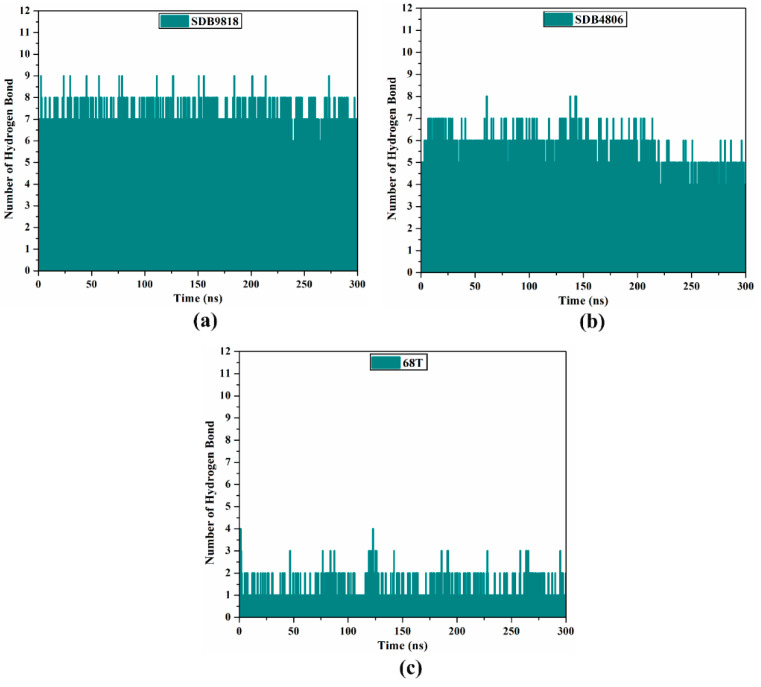
H-bond number for (**a**) SDB9818, (**b**) SDB4806, and (**c**) 68T bound to the allosteric site of DENV2 RdRp throughout 300 ns MDS.

The steadiness and binding affinity of inhibitors with the receptor were investigated by gauging the number of conventional H-bonds established during the MDS. Figure 9 portrays the number of H-bonds between the investigated streptomycetes NPs and the key residues of the DENV2 RdRp allosteric site throughout 300 ns MDS. As shown in Figure 9, the average number of H-bonds was 7, 6, and 2 for SDB9818-, SDB4806-, and **68T**-RdRp complexes, respectively. Notably, the average number of H-bonds for the identified streptomycetes NPs was more remarkable compared to the co-crystallized **68T** complexed with the DENV2 RdRp, indicating the higher stability of these streptomycetes NPs than **68T** within the DENV2 RdRp allosteric site over 300 ns MDS. These results firmly endorsed the anticipated H-bond interactions between the investigated streptomycetes NPs and the allosteric site amino acids of RdRp via molecular docking computations.

### 2.5. Physicochemical Characteristics

The drug-likeness of a molecule is estimated in accordance with Lipinski’s rule of five (Ro5), which detects whether the compound is orally active or not. Table 2 lists the drug-likeness features of the identified streptomycetes NPs and **68T**. According to Ro5, a molecule failing to meet more than two of its criteria is likely to have poor absorption in preclinical testing [59]. According to data listed in Table 2, SDB9818 violates only the HBD limit (6 vs. 5), which may marginally impact permeability but not necessarily disqualify it as a lead, especially given its favorable solubility (Log*P* = 0.40). SDB4806 slightly exceeds the MW threshold (511.52 g/mol) but remains within acceptable bounds for lead optimization. The Log*P* and H-bond of SDB4806 counts were favorable. **68T** complied fully with Ro5, supporting its drug-like profile. More precisely, the MW was in the range of 423.46 to 511.52 g/mol for the investigated streptomycetes NPs and **68T**. Moreover, the HBD was 6, 4, and 2 for SDB9818, SDB4806, and **68T**, respectively. SDB4806, SDB9818, and **68T** were found to have a number of HBA with values ranging from 7 to 10. The log*P* was less than 5 for the investigated streptomycetes NPs and **68T**. Notably, these results underscore the promise of the identified streptomycetes NPs as potential allosteric inhibitors of DENV2 RdRp and present them as viable candidates for future experimental validation.

**Figure 10:**
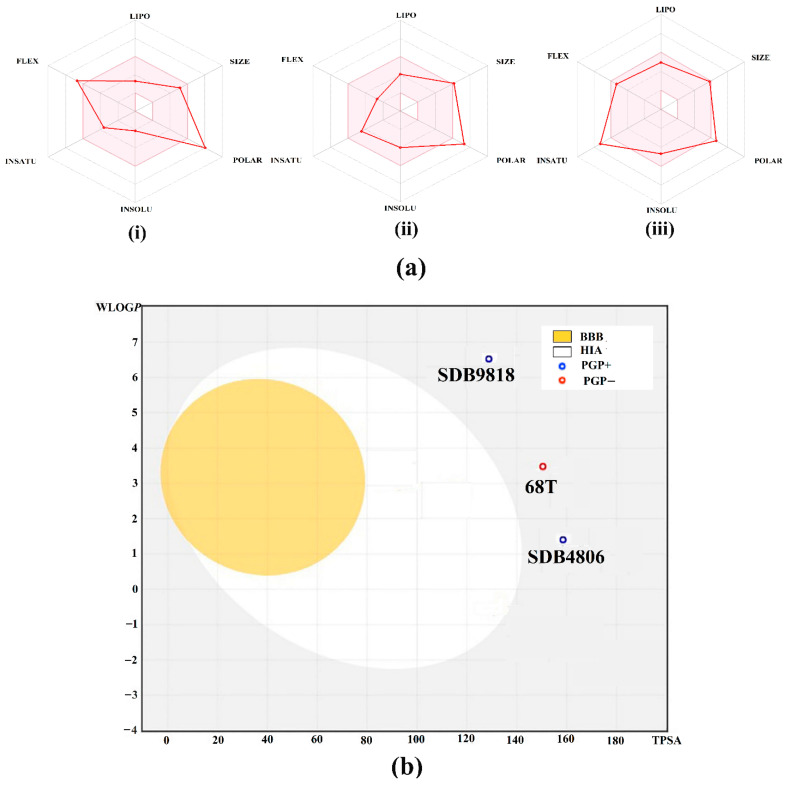
(**a**) Physicochemical radars for (i) SDB4806, (ii) SDB9818, and (iii) **68T** illustrating six key ADME-relevant properties: lipophilicity (LIPO), size, polarity (POLAR), solubility (INSOLU), flexibility (FLEX), and saturation (INSATU) and (**b**) boiled-egg model generated to predict gastrointestinal absorption (white region) and blood–brain barrier (BBB) permeability (yellow region) for the identified streptomycetes NPs and **68T** as prospective anti-DENV2 drug candidates.

Figure 10a displays the bioavailability radar plots of the identified streptomycetes NPs and **68T** and their drug-likeness characteristics. The pink area inside the hexagonal shape represents the ideal range for the investigated compounds. The recommended parameters for the drug-likeness of a small molecule are as follows: (i) insaturation (INSITU): a fraction of carbons exhibiting *sp*^3^ hybridization of at least 0.25; (ii) insolubility (INSOLE): a log *S* not exceeding 6; (iii) hydrophobicity (LIPO): ranging from −0.7 to +5.0; (iv) rotatable bonds (FLEXI): a maximum of 9 rotatable bonds; (v) molecular weight (SIZE): between 150 and 500 g/mol; and (vi) polar surface area (POLAR): between 20 and 130 g/mol, along with polar surface area (POLAR): within the range of 20 and 130 Å^2^. According to data presented in Figure 10a, the investigated streptomycetes NPs and **68T** lie within the passable range, indicating their favorable drug-like characteristics.

In addition, the pharmacokinetic features of the identified streptomycetes NPs and **68T** were investigated using a BOILED-Egg model (Figure 10b). The BOILED-Egg model demonstrates its utility in simultaneously forecasting two significant pharmacokinetic properties, specifically, passive gastrointestinal absorption (HIA) and penetration through the blood–brain barrier (BBB). As shown in Figure 10b, SDB9818, SDB4806, and **68T** were detected in the grey region, indicating that these compounds demonstrated low passive HIA and BBB. Consequently, the investigated compounds would gain better bioavailability profiles during a drug development process.

### 2.6. QM Computations

**Figure 11:**
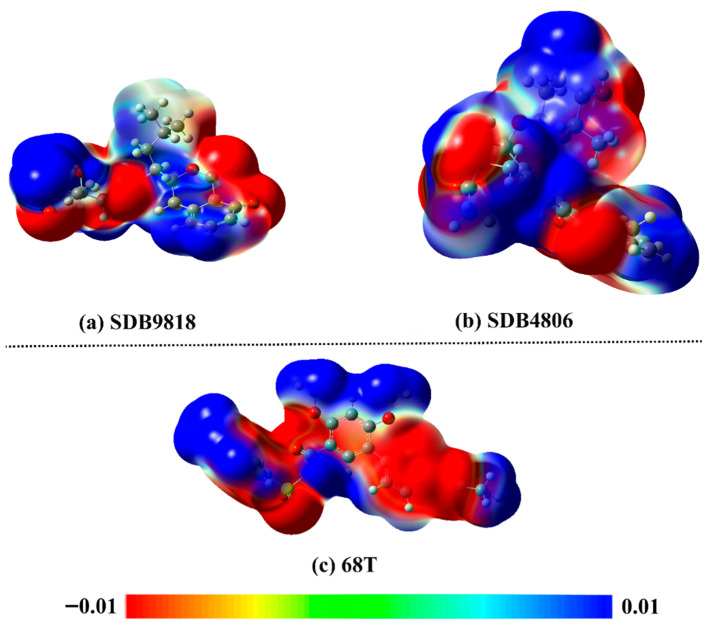
MEP maps of the last snapshot of (**a**) SDB9818, (**b**) SDB4806, and (**c**) **68T**.

ESP analysis is a reliable approach to demonstrate the negative and positive potentials on the surface of a chemical molecule. For the optimized last snapshot of streptomycetes NPs and **68T** retrieved from MDS, the MEP maps were generated and are graphed in Figure 11. As delineated in Figure 11, red regions were noticed above the N and O atoms of the investigated streptomycetes NPs and **68T**, indicating their nucleophilic nature. Additionally, blue regions were marked above the H atoms of the inspected streptomycetes NPs and **68T**, implying their electrophilic nature. According to the MEP maps, streptomycetes NPs and **68T** displayed the capability of forming H-bonds with fundamental residues within the allosteric site of DENV2 RdRp.

**Figure 12:**
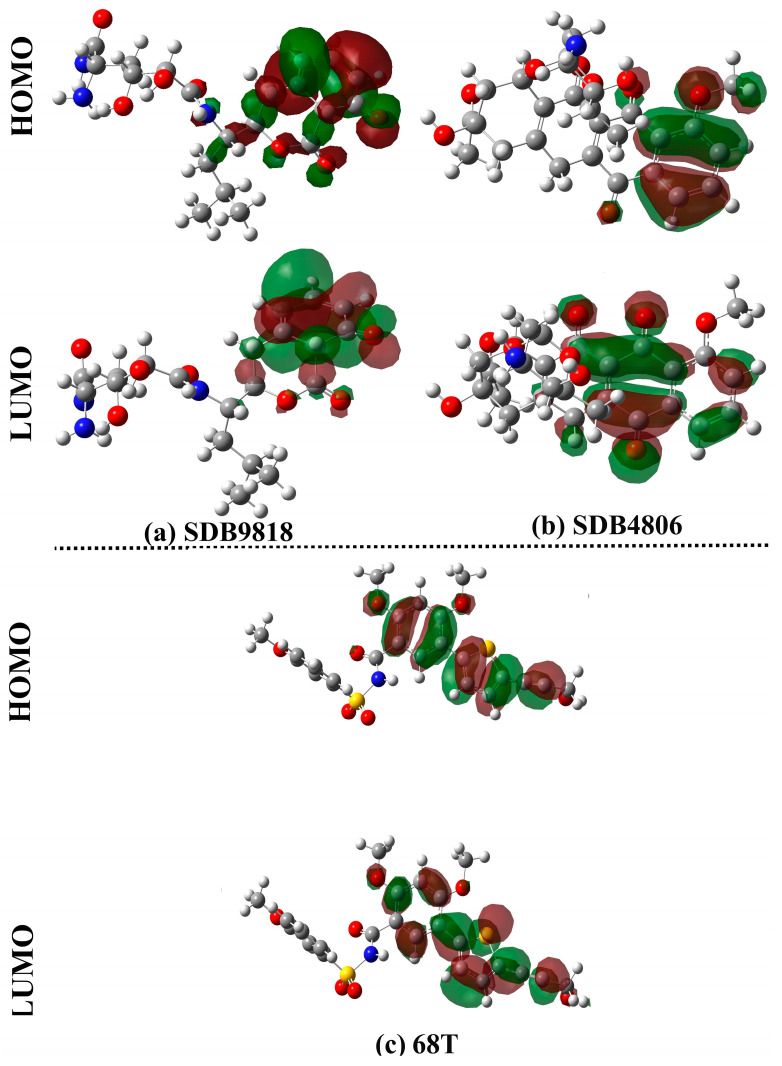
The distribution of HOMO and LUMO of the last snapshot of (**a**) SDB9818, (**b**) SDB4806, and (**c**) **68T**.

Based on Figure 12, HOMO levels were mainly found around the electron-rich zones of the investigated compounds (e.g., O and N atoms). In addition, LUMO levels were concentrated around the electron-deficient regions in the investigated streptomycetes NPs and 68T (e.g., H atoms). As numerical evidence from Table 3, the *E*_HOMO_/*E*_LUMO_ values were −7.99/−1.34, −8.06/−2.33, and −6.88/−0.83 eV for SDB9818, SDB4806, and **68T**, respectively. Moreover, SDB9818, SDB4806, and **68T** revealed *E*_FL_ values in the range of −3.85 to −5.19 eV. SDB9818, SDB4806, and **68T** displayed low *E*_gap_ with values of 6.65, 5.74, and 6.05 eV, respectively. Of note, the low *E*_gap_ values indicated the considerable chemical reactivity of the investigated streptomycetes NPs and **68T**.

According to the indisputable role of electronic parameters, global descriptors were computed for SDB9818, SDB4806, and **68T** (Table 4). As reported in Table 4, SDB9818, SDB4806, and **68T** demonstrated *IP* values ranging from 6.88 to 8.71 eV. In addition, the *EA* values were found to be 1.34, 2.33, and 0.83 eV for SDB9818, SDB4806, and **68T**, respectively. The *η* and *S* of the investigated compounds can serve as an indicator for their stability and chemical reactivity. The *η* values for SDB9818, SDB4806, and **68T** ranged from 2.87 to 3.32 eV. SDB9818, SDB4806, and **68T** unveiled outstanding *S* values of 0.30, 0.35, and 0.33 eV^−1^, respectively. Among the three compounds, SDB4806 exhibited the highest softness (0.35 eV^−1^), suggesting a greater capacity for electronic adaptability. Generally, the obtained *η* and *S* values of SDB9818, SDB4806, and **68T** indicated promising polarizability and electron-donating/accepting flexibility, which facilitated effective interactions with DENV RdRp allosteric site through non-covalent interactions (e.g., H-bonds, electrostatic interactions, and π–π stacking). Moreover, the computed global descriptors provided an electronic rationale for the biological activity of the investigated compounds and reinforced their potential as effective inhibitors for the DENV2 RdRp allosteric site.

## 3. Computational Methodology

### 3.1. RdRp Preparation

The X-ray crystallographic structure of DENV2 RdRp (PDB entry: 5K5M, resolution: 2.1 Å) was chosen and employed as a template for all *in silico* computations [41]. All heteroatoms, including ions, inhibitors, and water molecules, were extracted for preparation purposes. The ionization status of titratable residues at a pH of 7.4 was determined utilizing the PropKa3 software [60]. All missing H-atoms were consequently inserted.

### 3.2. Streptome Database Preparation

The Streptome database, involving > 6500 NPs derived from approximately 3300 streptomycetes strains, was retrieved in SDF format [61]. Duplicates were removed in accordance with the International Chemical Identifier (InChIKey) [62]. Omega2 software (version 4.1.1.0) was utilized for generating 3D structures for each streptomycete NP [63,64]. All generated 3D structures were then adopted for an optimization process utilizing the MMFF94S force field implemented in SZYBKI software (version 2.4.0.0) [65,66]. The FixPka tool within the QUACPAC program (version 2.1.3.0) was used to determine the dominant ionization state at a pH of 7.4 [67]. The atomic charges of the streptomycetes NPs were computed with the assistance of the Gasteiger-Marsili method [68]. At www.compchem.net/ccdb, accessed on 13 February 2024, all prepared streptomycetes NPs can be accessed.

### 3.3. Docking Computation

AutoDock4.2.6 software was applied to accomplish molecular docking computations [69]. For docking calculations, the DENV2 RdRp enzyme was saved in the pdbqt format with the help of MGLTools 1.5.7 [70]. This research included two stages of docking calculations, namely standard and expensive docking computations. For standard and expensive docking computations, the genetic algorithm (*GA*) runs were set to 50 and 250 iterations, respectively. Moreover, 5 and 25 million energy evaluations (*eval*) were adjusted to standard and expensive docking computations, respectively. Other docking parameters were left at their defaults. The grid was designed to include the entire allosteric site of the DENV2 RdRP, measuring 40 × 40 × 40 Å^3^, with a spacing value of 0.375 Å. Grid coordinates were located at *x* = −15.551, *y* = −44.03, and *z* = −19.247. The docking conformation with the highest binding affinity was selected from the largest cluster as a representative docking mode.

### 3.4. MD Simulations (MDS)

All MDS for the most promising streptomycetes NPs complexed with DENV2 RdRp allosteric site were accomplished utilizing AMBER20 software [71]. Parameters for conducting MDS are more fully described elsewhere [72,73,74]. Briefly, DENV2 RdRp was characterized by the AMBER force field 14SB [75]. The general AMBER Force Field (GAFF2) was utilized for the parameterization of the inspected streptomycetes NPs [76]. Using Gaussian09 software, the geometrical optimization for the inspected streptomycetes NPs was executed at the HF/6-31G* level [77]. The restrained electrostatic potential (RESP) was then used for computing the atomic charges for the optimized streptomycetes NPs [78]. In a truncated octahedral periodic box, each RdRp-NP complex was solvated with the TIP3P water model [79]. In order to preserve the electroneutrality of the inspected RdRp-NP complexes, sodium or chloride counterions were added. The isosmotic salt environment was also maintained by the insertion of 0.15 M NaCl. The inspected complexes were advanced for 5000 iterations of energy minimization. Following that, the investigated complexes were gently annealed up to 310 K throughout 50 ps. The heated complexes were submitted to the equilibration stage for 10 ns. Finally, the production runs were carried out on the equilibrated complexes throughout 10, 200, and 300 ns. MD trajectories were recorded every 10 ps for post-MD analyses, which were performed using the CPPTRAJ tool [80]. MDS was accelerated using the PMEMD version of CUDA on GPU cores. All molecular interactions were displayed utilizing the Discovery Studio module of Biovia software (version 2019) [81].

### 3.5. Binding Energy Computations

To evaluate the binding energy between DENV2 RdRp and streptomycetes NPs, the molecular mechanics/generalized Born surface area (MM/GBSA) approach was used [82]. The binding energy was estimated by the following equation:

(1) $${\Delta G}_{binding}=G_{RdRp-NP\;}-G_{RdRp}\;-G_{NP}$$

where the energy term (*G*) was numerically assessed as follows:

(2) $$G=E_{vdW}+G_{SA}+G_{GB\;}+E_{ele}-TS$$

*E*_vdW_ points out van der Waals energy. *G*_SA_ and *G*_GB_ imply non-polar and polar contributions of the desolvation energy, respectively. Using the modified GB model (iGB = 2) developed by Onufriev et al., the *G*_GB_ was computed [83]. *E*_ele_ stands for electrostatic energy. T indicates absolute temperature, and *S* is the entropy participation. Entropic participation was not considered due to its high computation costs [84,85]. Notably, the exclusion of the entropic component had no significant effect on the MM/GBSA binding energy estimations [86].

### 3.6. Physicochemical Features

The SwissADME web server was employed to estimate the physicochemical characteristics of the identified streptomycetes NPs based on Lipinski’s rule of five (Ro5) conditions [87]. Lipinski’s rule states that (i) the number of H-bond acceptors (HBA) of the investigated compound should be <10, (ii) the molecular weight (MW) should not be >500 daltons, (iii) the number of H-bond donors (HBD) should be <5, and (iv) the partition coefficient (log*P*) should not be >5. In addition, the BOILED-Egg method was utilized for predicting gastrointestinal absorption and brain permeation of the identified streptomycetes NPs [88]. In the shape of a boiled egg, the plot is composed of white, grey, and yolk regions. More exactly, compounds in white zones are those that are more likely to be absorbed by the gastrointestinal tract, while compounds in yolk zones are those that are more likely to permeate the brain. Meanwhile, compounds in grey zones are those that are less likely to be non-absorbed by the gastrointestinal tract or non-brain permeating. For dot coloring, the blue dots indicate that the compound could be effluxed from the Central Nervous System (CNS) by P-Glycoprotein (PGP), whereas the red dots mean that compounds could not be effluxed from CNS by PGP [88].

### 3.7. Quantum Mechanical (QM) Computations

In the realm of QM computations, the final frame of the identified streptomycetes NPs elicited from MDS was geometrically optimized utilizing the M062X/6-311+G** level of theory with the assistance of Gaussian09 software [77]. For the optimized streptomycetes NPs, electrostatic potential (ESP) analysis was performed, and the corresponding molecular electrostatic potential (MEP) maps were generated at an electron density envelope of 0.002 au [89]. The frontier molecular orbitals (FMOs) theory was implemented for a deeper understanding of the electronic features of the optimized streptomycetes NPs. As a result, the energetic values and electronic patterns of the highest occupied/lowest unoccupied molecular orbital (i.e., HOMO/LUMO) were generated. Furthermore, the Fermi level (*E*_FL_) energy and energy gap (*E*_gap_) were computed on the basis of *E*_LUMO_ and *E*_HOMO_, as shown in Equations (3) and (4), respectively.

(3) $$E_{FL}=E_{HOMO}+\frac{E_{LUMO}-E_{HOMO}\;}{2}$$

(4) $$E_{gap}=E_{LUMO}-E_{HOMO}$$

As well, further electronic parameters such as global hardness (*η*), global softness (*S*), ionization potential (*IP*), and electron affinity (*EA*) were computed as follows:

(5) $$\eta =\frac{E_{LUMO}-E_{HOMO}\;}{2}$$

(6) $$IP=-E_{HOMO}$$

(7) $$S=\frac{1}{\eta }$$

(8) $$EA=-E_{LUMO}$$

## 4. Conclusions

Recently, the dengue virus, a commonly encountered flavivirus, has shown a range of epidemiological, economic, and health effects globally. DENV2 is a significant factor in mortality associated with dengue. DENV2 RdRp is an attractive druggable target owing to its essential role in the replication of the virus. In the present work, the Streptome database, containing > 6500 compounds, was mined to hunt the most prospective inhibitors against the allosteric site of RdRp, utilizing advanced *in silico* approaches. Based on docking computations and MDS coupled with the MM/GBSA binding energy evaluations, SDB4806 and SDB9818 revealed promising binding affinities with Δ*G*_binding_ < −188.3 kJ.mol^−1^ against RdRp. The constancy of the identified streptomycetes NPs bound to the DENV2 RdRp allosteric site was confirmed upon the post-MD analyses throughout 300 ns. Based on the drug-likeness features, the identified streptomycetes NPs unveiled good oral bioavailability. Quantum mechanical computations were also performed for the identified streptomycetes NPs, and the outcomes demonstrated their stability and chemical reactivity. These findings provide a solid basis for future experimental validations and in-vitro studies aimed at combating DENV2.

## Figures and Tables

**Table 1:** 2D Chemical structures, computed docking scores, and intramolecular H-bond of the top 10 scoring streptomycetes NPs toward the DENV2 RdRp allosteric site ^a^.

Compound Name/ID	2D Chemical Structure	Docking Score (kJ.mol^−1^)		Intermolecular H-Bond
		Standard	Expensive	
68T	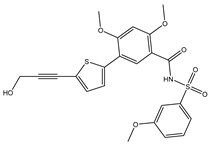	−35.6	−35.6	ARG729 (3.11 Å), TRP795 (3.40 Å), GLU802 (2.67 Å)
SDB9818	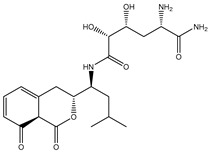	−46.9	−46.9	ARG737 (2.04; 2.62; 1.83 Å), SER796 (3.01 Å), CYS709 (1.83 Å), ASP664 (1.74 Å), TRP795 (1.80 Å), SER710 (1.70 Å)
SDB4806	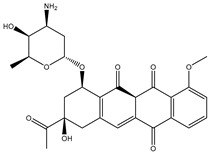	−42.7	−45.6	GLU459 (1.63 Å), ASP664 (1.84 Å), ARG729 (2.16 Å), ARG737 (2.84 Å), THR794 (1.97; 2.44 Å), TRP795 (2.54 Å), SER796 (2.83 Å)
SDB895	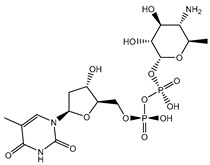	–41.8	–45.2	LYS461 (2.90 Å), ASP664 (1.81 Å), ARG737 (3.72 Å), SER796 (2.22; 2.33; 2.08 Å),
SDB12947	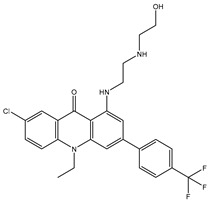	–35.6	–45.2	ASP664 (1.88 Å), HIS798 (3.18 Å), SER796 (3.02; 3.18 Å), CYS709 (2.19 Å)
SDB13026	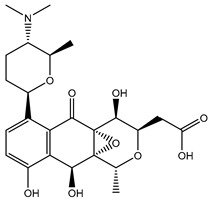	–46.9	–45.2	LYS461 (2.09; 2.12; 2.22 Å), ASP664 (2.45 Å), ARG729 (2.33; 2.35 Å), TYR766 (2.83 Å), ARG737 (1.73 Å),
SDB9891	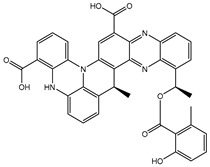	–42.3	–45.2	LYS461 (1.90 Å), SER796 (1.72; 2.25; 3.15 Å), TYR766 (2.02; 2.15 Å), HIS798 (3.08 Å), CYS709 (1.83 Å)
SDB10285	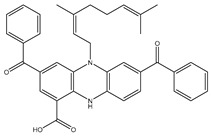	–41.8	–42.3	LYS461 (2.24 Å), ARG472 (2.95 Å), ARG737 (2.53 Å), GLU802 (1.86; 2.36 Å)
SDB993	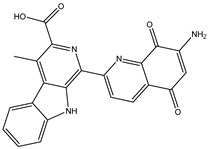	–41.8	–42.3	SER796 (2.65 Å), ARG737 (2.01 Å)
SDB1014	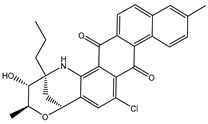	–41.4	–40.2	LYS461 (2.60 Å), SER796 (2.84 Å)
SDB827	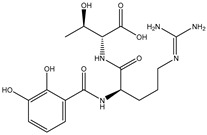	–41.4	–39.3	ASP664 (2.13; 2.23 Å), ARG729 (3.07 Å), ARG737 (1.99, 2.57 Å), TYR766 (2.09, 2.01 Å), THR794 (2.41 Å), SER796 (2.12, 2.19 Å)

^a^ Streptomycetes NPs were ordered according to the expensive docking computations.

**Table 2:** The drug-like characteristics of the identified streptomycetes NPs and **68T** as prospective anti-DENV2 drug candidates.

Compound Name/ID	Log*P*	MW (g/mol)	HBD	HBA
**68T**	4.23	487.55	2	7
SDB9818	0.40	423.46	6	8
SDB4806	2.24	511.52	4	10

**Table 3:** The computed *E*_HOMO_, *E*_LUMO_, *E*_FL_, and *E*_gap_ in eV for the most promising streptomycetes NPs and **68T**.

Compound Name/ID	*E*_HOMO_	*E*_LUMO_	*E*_FL_	*E*_gap_
**68T**	−6.88	−0.83	−3.85	6.05
SDB9818	−7.99	−1.34	−4.67	6.65
SDB4806	−8.06	−2.33	−5.19	5.74

**Table 4:** Computed global descriptors for SDB4806, SDB9818, and **68T**.

Compound Name/ID	*IP* (eV)	*EA* (eV)	*η* (eV)	*S* (eV^−1^)
**68T**	6.88	0.83	3.03	0.33
SDB9818	7.99	1.34	3.32	0.30
SDB4806	8.06	2.33	2.87	0.35

## Data Availability

The data presented in this study are available in the Supplementary Material.

## Supplementary Materials

The following supporting information can be downloaded at https://www.mdpi.com/article/10.3390/ph18081135/s1: Figure S1: 2D molecular interactions of the anticipated binding modes for the top 39 streptomycetes NPs against the allosteric site of DENV2 RdRp; Figure S2: Overlapping between the predicted docking poses using a *GA* of 250 (pink) and 1000 (cyan) of (**a**) SDB9818 and (**b**) SDB4806 inside the DENV2 RdRp allosteric site. The computed docking score is displayed in kJ.mol^−1^; Figure S3: 3D binding patterns of (**a**) SDB9818 and (**b**) SDB4806 inside the DENV2 RdRp allosteric site at time intervals of 50, 100, 150, 200, 250, and 300 ns MDS; Table S1: The anticipated standard and expensive docking scores (in kJ.mol^−1^) for the top 151 streptomycetes NPs and **68T** toward DENV2RdRp allosteric site; Table S2: Estimated standard and expensive docking scores and MM/GBSA binding energies (in kJ.mol^−1^) over 10 ns MD simulations of the promising 39 streptomycetes NPs toward DENV2 RdRp allosteric site; Table S3: Estimated MM/GBSA binding energies, standard deviation, and standard error of the mean (in kJ.mol^−1^) over 300 ns MDS of the most promising two streptomycetes NPs and 68T toward the DENV2 RdRp allosteric site.
