# Supplementary material for: In Silico Mining of the Streptome Database for Hunting Putative Candidates to Allosterically Inhibit the Dengue Virus (Serotype 2) RdRp
Source: Pharmaceuticals (Basel). 2025 Jul 30;18(8):1135. doi: 10.3390/ph18081135 (PMC12389343; doi:10.3390/ph18081135)
Supplement: Supplementary file 1 [file pharmaceuticals-18-01135-s001.zip › pharmaceuticals-3724877-supplementary.pdf]

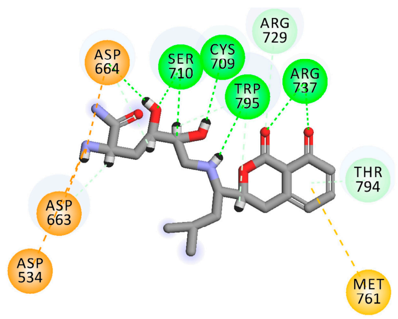

SDB9818

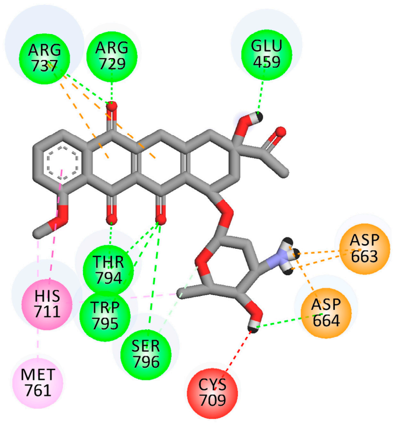

SDB4806

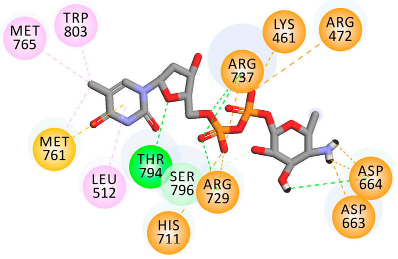

SDB895

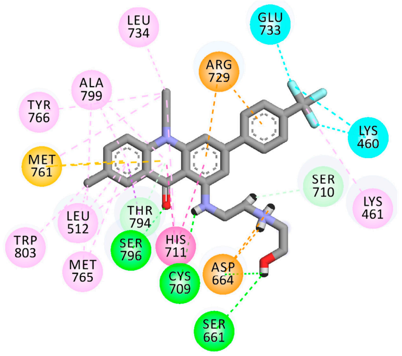

SDB12947

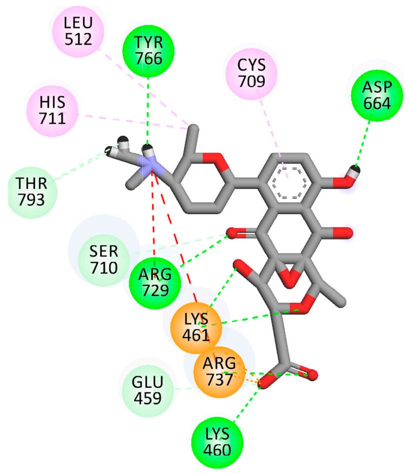

SDB13026

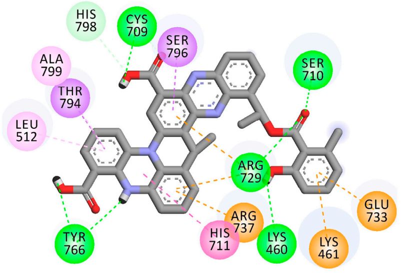

SDB9891

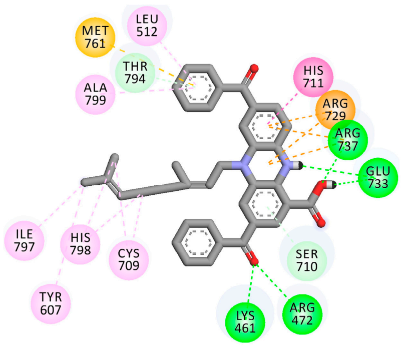

SDB10285

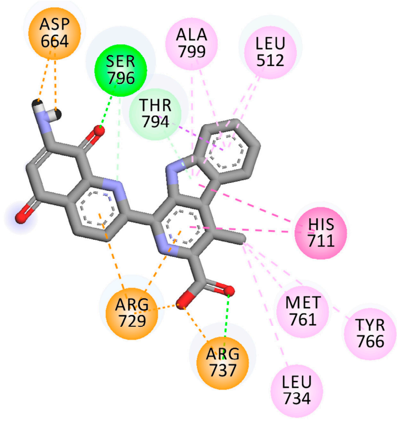

SDB993

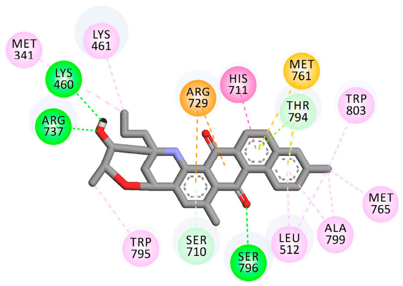

SDB1014

Figure S1. 2D molecular interactions of the anticipated binding modes for the top 39 streptomycetes NPs against the allosteric site of DENV2 RdRp.

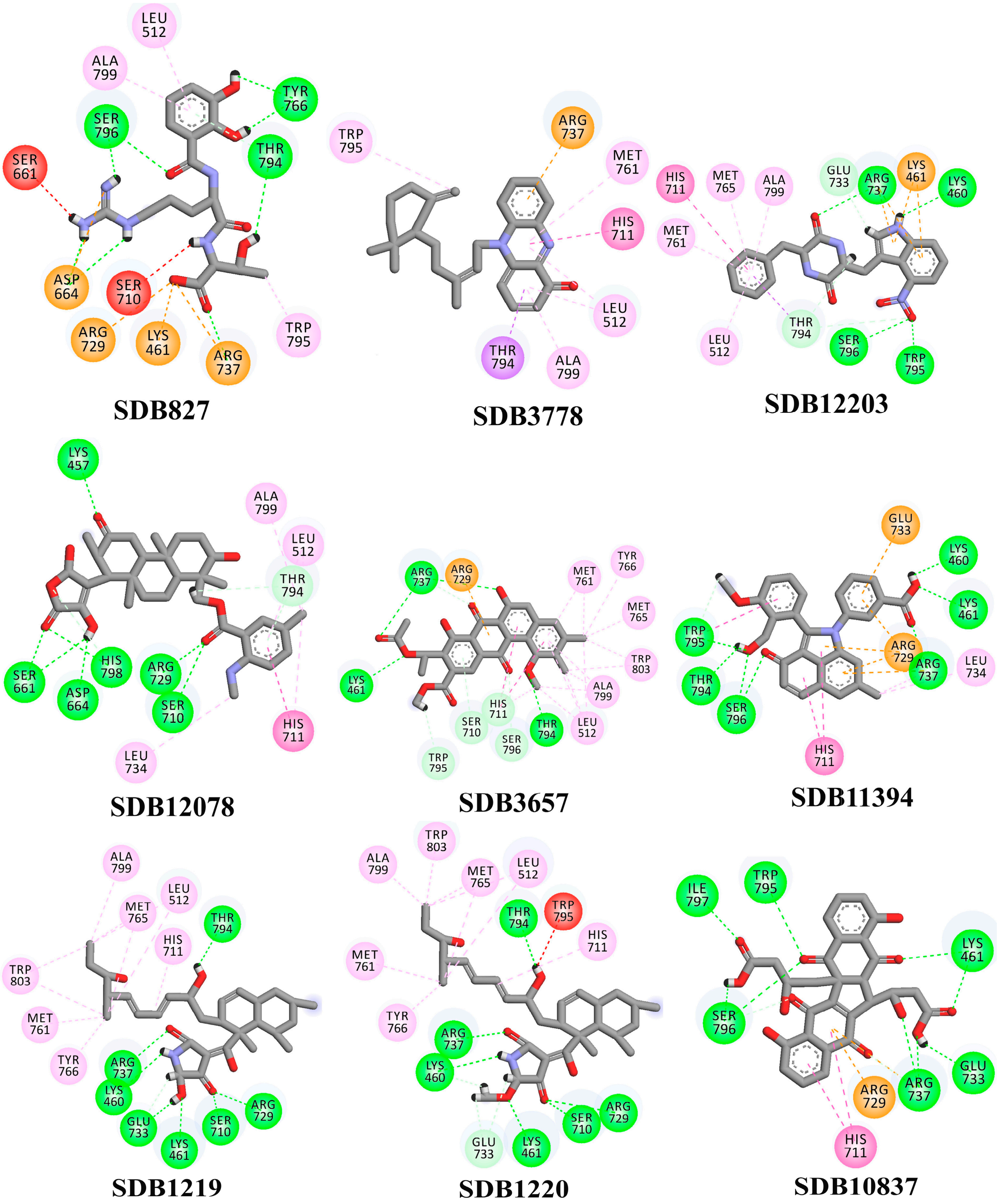

Figure S1. *Continued.*

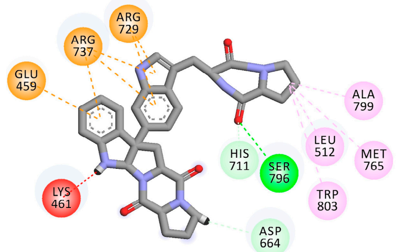

SDB10935

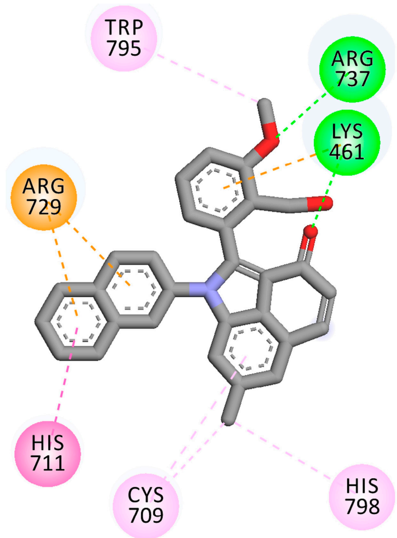

SDB11385

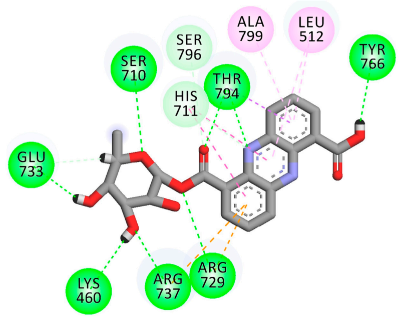

SDB10332

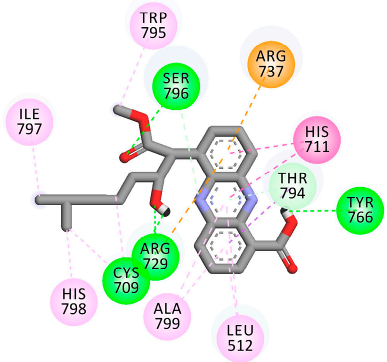

SDB10240

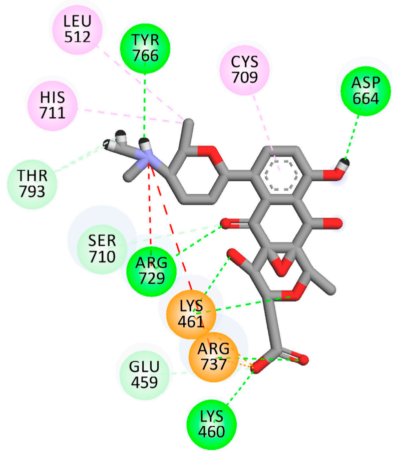

SDB13026

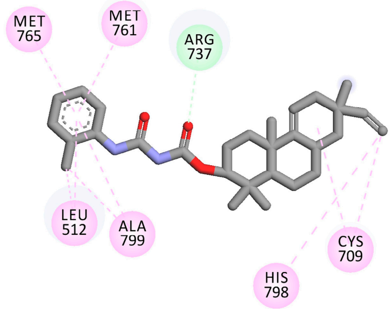

SDB13172

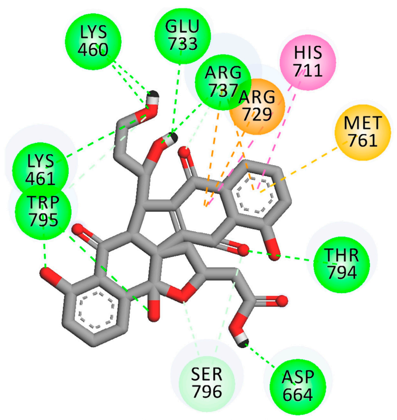

SDB10838

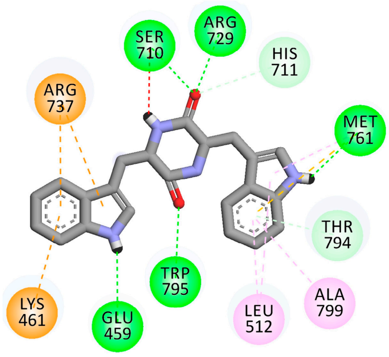

SDB9935

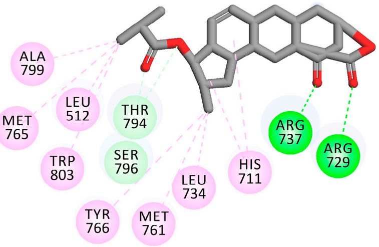

SDB9474

Figure S1. *Continued.*

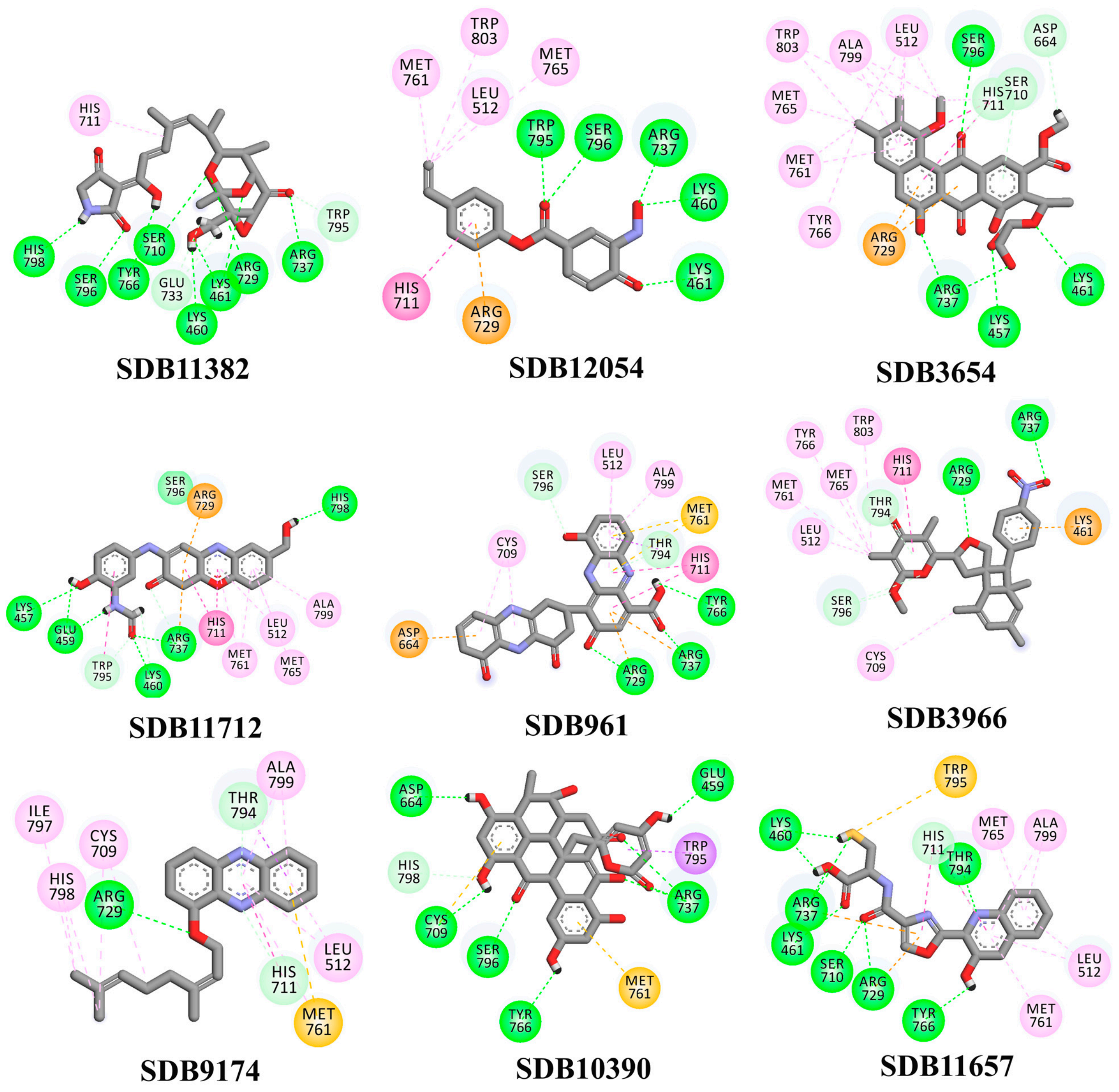

Figure S1. *Continued.*

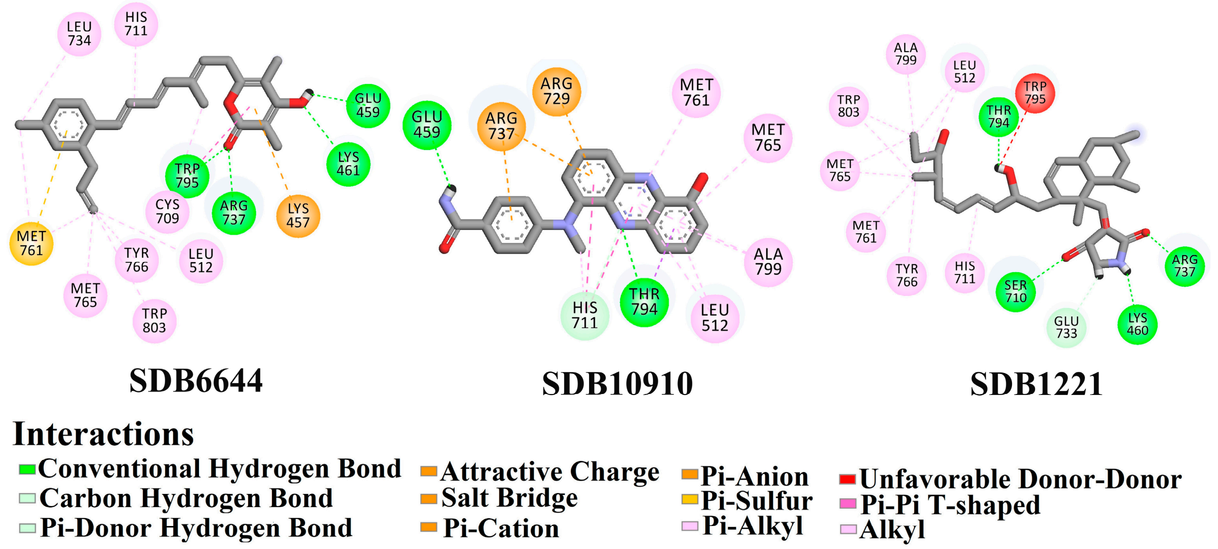

Figure S1. *Continued.*

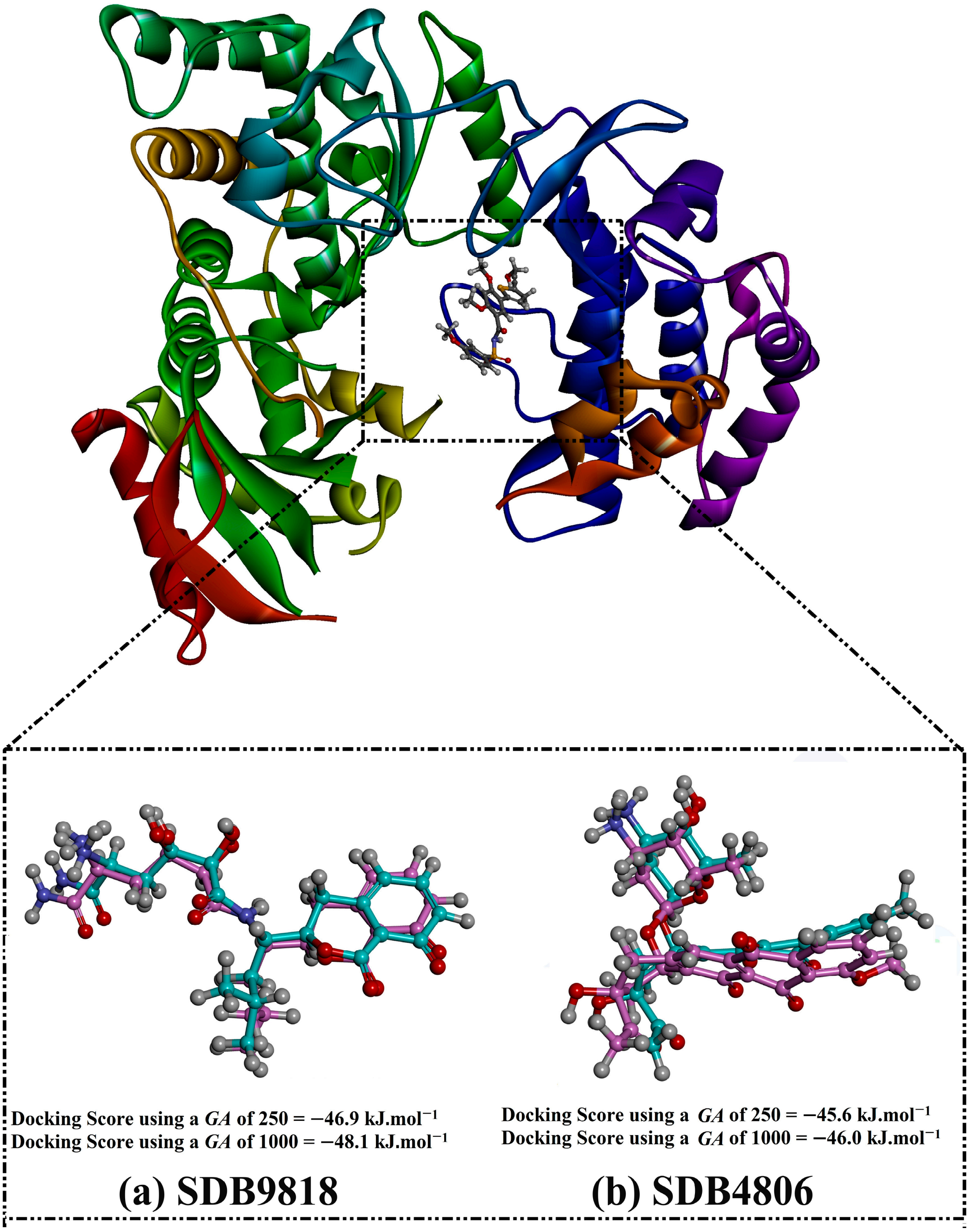

Figure S2. Overlapping between the predicted docking poses using a *GA* of 250 (pink) and 1000 (cyan) of (a) SDB9818 and (b) SDB4806 inside the DENV2 RdRp allosteric site. The computed docking score is displayed in kJ.mol^{-1}.

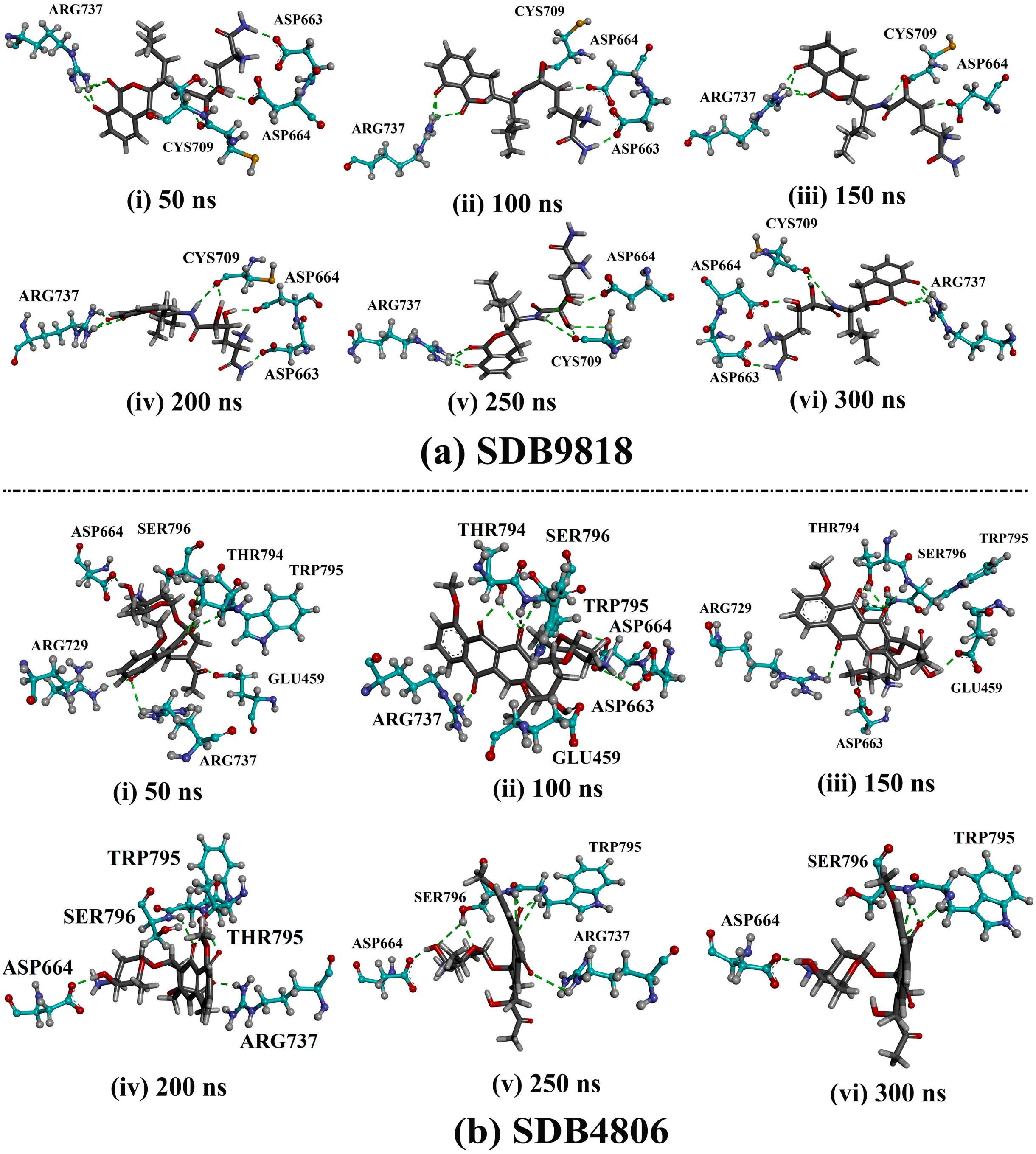

Figure S3. 3D binding patterns of (a) SDB9818 and (b) SDB4806 inside the DENV2 RdRp allosteric site at time intervals of 50, 100, 150, 200, 250, and 300 ns MDS.

Table S1. The anticipated standard and expensive docking scores (in kJ.mol^{-1}) for the top 151 streptomycetes NPs and **68T** towards DENV2RdRp allosteric site [a].

No.	Compound Code	Docking Score (kJ.mol^{-1})		No.	Compound Code	Docking Score (kJ.mol^{-1})	
		Standard	Expensive			Standard	Expensive
	**68T**	**–35.6**	**–35.6**	44	SDB6646	–35.6	–35.1
1	SDB9818	–46.9	–46.9	45	SDB9212	–35.6	–35.1
2	SDB4806	–42.7	–45.6	46	SDB10911	–35.6	–35.1
3	SDB895	–41.8	–45.2	47	SDB10180	–35.6	–35.1
4	SDB12947	–35.6	–45.2	48	SDB11681	–35.6	–35.1
5	SDB13026	–46.9	–45.2	49	SDB6663	–35.6	–35.1
6	SDB9891	–42.3	–45.2	50	SDB4504	–35.6	–35.1
7	SDB10285	–41.8	–42.3	51	SDB6406	–35.6	–35.1
8	SDB993	–41.8	–42.3	52	SDB11714	–35.6	–34.7
9	SDB1014	–41.4	–40.2	53	SDB36	–35.6	–34.7
10	SDB827	–41.4	–39.3	54	SDB9417	–35.6	–34.7
11	SDB3778	–41.4	–39.3	55	SDB9715	–35.6	–34.7
12	SDB12203	–39.3	–39.3	56	SDB13712	–35.6	–34.7
13	SDB12078	–38.5	–38.9	57	SDB12927	–35.6	–34.7
14	SDB3657	–38.1	–38.5	58	SDB13330	–35.6	–34.7
15	SDB11394	–38.1	–38.5	59	SDB3063	–35.6	–34.7
16	SDB1219	–37.7	–38.5	60	SDB5009	–35.6	–34.7
17	SDB1220	–37.7	–38.5	61	SDB10928	–35.6	–34.7
18	SDB10837	–37.2	–37.2	62	SDB3315	–35.6	–34.7
19	SDB10935	–37.2	–37.2	63	SDB3656	–35.6	–34.7
20	SDB11385	–36.8	–37.2	64	SDB8976	–35.6	–34.7
21	SDB10332	–36.8	–37.2	65	SDB9175	–35.6	–34.7
22	SDB10240	–36.8	–37.2	66	SDB10265	–35.6	–34.7
23	SDB13026	–36.8	–36.8	67	SDB968	–35.6	–34.3
24	SDB13172	–36.8	–36.8	68	SDB9264	–35.6	–34.3
25	SDB10838	–36.4	–36.8	69	SDB11400	–35.6	–34.3
26	SDB9935	–36.4	–36.8	70	SDB13243	–35.6	–34.3
27	SDB9474	–36.4	–36.8	71	SDB9756	–35.6	–34.3
28	SDB11382	–36.4	–36.4	72	SDB9983	–35.6	–35.1
29	SDB12054	–36.4	–36.4	73	SDB5178	–35.6	–35.1
30	SDB3654	–36.0	–36.4	74	SDB10378	–35.6	–35.1
31	SDB11712	–36.0	–36.0	75	SDB6646	–35.6	–35.1
32	SDB961	–36.0	–36.0	76	SDB9212	–35.6	–35.1
33	SDB3966	–36.0	–36.0	77	SDB10911	–35.6	–35.1
34	SDB9174	–36.0	–36.0	78	SDB10180	–35.6	–35.1
35	SDB10390	–36.0	–36.0	79	SDB11681	–35.6	–35.1
36	SDB11657	–36.0	–35.6	80	SDB6663	–35.6	–34.7
37	SDB6644	–35.6	–35.6	81	SDB4504	–35.6	–34.7
38	SDB10910	–35.6	–35.6	82	SDB6406	–35.6	–34.7
39	SDB1221	–35.6	–35.6	83	SDB11714	–35.6	–34.7
40	SDB10377	–35.6	–35.1	84	SDB36	–35.6	–34.7
41	SDB11381	–35.6	–35.1	85	SDB9417	–35.6	–34.7
42	SDB9538	–35.6	–35.1	86	SDB9715	–35.6	–34.7
43	SDB11392	–35.6	–35.1	87	SDB13712	–35.6	–34.7

Table S1. *Continued.*

No.	Compound Code	Docking Score (kJ.mol^{-1})		No.	Compound Code	Docking Score (kJ.mol^{-1})	
		Standard	Expensive			Standard	Expensive
88	SDB11401	–35.6	–8.2	121	SDB13661	–34.3	–33.5
90	SDB10485	–35.6	–8.2	122	SDB11483	–34.3	–33.5
91	SDB11687	–35.6	–8.2	123	SDB3717	–34.3	–33.5
92	SDB10404	–35.6	–8.1	124	SDB11780	–33.9	–33.1
93	SDB11455	–35.6	–8.1	125	SDB13591	–33.9	–33.1
94	SDB12792	–35.6	–8.1	126	SDB3804	–33.9	–33.1
95	SDB3823	–35.6	–8.1	127	SDB13659	–33.9	–33.1
96	SDB5264	–35.6	–8.1	128	SDB824	–33.9	–33.1
97	SDB11387	–35.6	–8.1	129	SDB9426	–33.9	–33.1
98	SDB13711	–35.6	–8.1	130	SDB11402	–33.9	–33.1
99	SDB11398	–35.6	–8.1	131	SDB9285	–33.9	–33.1
100	SDB12170	–35.6	–8.1	132	SDB10807	–33.9	–33.1
101	SDB3118	–35.6	–8.1	133	SDB10441	–33.9	–33.1
102	SDB5152	–35.6	–8.1	134	SDB10251	–33.9	–33.1
103	SDB11130	–35.6	–8.1	135	SDB12154	–33.9	–33.1
104	SDB11388	–35.6	–8.1	136	SDB11396	–33.9	–32.6
105	SDB11386	–35.6	–8.1	137	SDB11383	–33.9	–32.6
106	SDB13710	–35.6	–8.1	138	SDB11748	–33.9	–32.6
107	SDB3764	–35.6	–8.1	139	SDB5019	–33.9	–32.6
108	SDB8928	–35.6	–8.1	140	SDB10367	–33.9	–32.6
109	SDB8840	–35.6	–8.0	141	SDB10578	–33.5	–32.2
110	SDB9286	–35.6	–8.0	142	SDB10923	–33.5	–32.2
111	SDB9331	–35.6	–8.0	143	SDB11399	–33.5	–32.2
112	SDB11415	–35.6	–8.0	144	SDB3021	–33.5	–31.4
113	SDB11731	–35.6	–8.0	145	SDB3562	–33.5	–31.4
114	SDB9767	–35.6	–8.0	146	SDB11397	–33.5	–31.0
115	SDB10034	–35.6	–8.0	147	SDB3718	–33.5	–30.5
116	SDB9037	–35.6	–8.0	148	SDB11389	–33.5	–30.1
117	SDB10657	–35.6	–8.0	149	SDB11490	–33.5	–30.1
118	SDB11690	–35.6	–8.0	150	SDB10590	–33.5	–30.1
119	SDB3847	–35.6	–8.0	151	SDB13173	–33.5	–30.1
120	SDB10227	–35.6	–8.0				

[a] Data were arranged according to expensive docking scores.

Table S2. Estimated standard and expensive docking scores and MM/GBSA binding energies (in kJ.mol^{-1}) over 10 ns MD simulations of the promising 39 streptomycetes NPs towards DENV2 RdRp allosteric site [a].

No.	Compound Code	Docking Score (kJ.mol^{-1})		MM/BSA Binding Energy (kJ.mol^{-1})
		Standard	Expensive	
	**68T**	**–35.6**	**–35.6**	**–167.8**
1	SDB4806	–42.7	–45.6	–246.4
2	SDB12947	–35.6	–45.2	–234.7
3	SDB9818	–46.9	–46.9	–228.4
4	SDB895	–41.8	–45.2	–209.6
5	SDB13026	–46.9	–45.2	–192.0
6	SDB1220	–37.7	–38.5	–166.9
7	SDB11657	–36.0	–35.6	–165.3
8	SDB10910	–35.6	–35.6	–158.6
9	SDB993	–41.8	–42.3	–156.5
10	SDB12078	–38.5	–38.9	–151.9
11	SDB9891	–42.3	–45.2	–150.6
12	SDB13026	–36.8	–36.8	–150.2
13	SDB10935	–37.2	–37.2	–146.9
14	SDB3778	–41.4	–39.3	–141.4
15	SDB1014	–41.4	–40.2	–138.5
16	SDB3966	–36.0	–36.0	–136.8
17	SDB827	–41.4	–39.3	–135.1
18	SDB10285	–41.8	–42.3	–132.2
19	SDB1219	–37.7	–38.5	–127.2
20	SDB9474	–36.4	–36.8	–123.4
21	SDB9935	–36.4	–36.8	–118.8
22	SDB11712	–36.0	–36.0	–116.7
23	SDB10837	–37.2	–37.2	–114.2
24	SDB13172	–36.8	–36.8	–113.0
25	SDB11385	–36.8	–37.2	–112.5
26	SDB961	–36.0	–36.0	–107.9
27	SDB10240	–36.8	–37.2	–104.2
28	SDB11382	–36.4	–36.4	–103.3
29	SDB11394	–38.1	–38.5	–102.9
30	SDB12054	–36.4	–36.4	–100.4
31	SDB3654	–36.0	–36.4	–97.9
32	SDB6644	–35.6	–35.6	–91.2
33	SDB3657	–38.1	–38.5	–89.1
34	SDB10332	–36.8	–37.2	–82.0
35	SDB10838	–36.4	–36.8	–79.5
36	SDB1221	–35.6	–35.6	–77.4
37	SDB9174	–36.0	–36.0	–71.5
38	SDB12203	–39.3	–39.3	–61.1
39	SDB10390	–36.0	–36.0	–54.4

[a] Data were arranged according to the MM/GBSA binding energy over 10 ns MD simulations.

Table S3. Estimated MM/GBSA binding energies, standard deviation, and standard error of the mean (in kJ.mol^{-1}) over 300 ns MDS of the most promising two streptomycetes NPs and 68T towards the DENV2 RdRp allosteric site.

Compound Name/Code	MM/BSA Binding Energy (kJ.mol^{-1})	Standard Deviation (kJ.mol^{-1})	Standard Error of the Mean (kJ.mol^{-1})
**68T**	−150.6	±14.8	0.09
SDB9818	−246.4	±20.0	0.12
SDB4806	−242.3	±28.0	0.16
